# Supplementary material for: Predicting Tick Presence by Environmental Risk Mapping
Source: Front Public Health. 2014 Nov 26;2:238. doi: 10.3389/fpubh.2014.00238 (PMC4244977; doi:10.3389/fpubh.2014.00238)
Supplement: Supplementary file 1 [file Presentation1.PDF]

Supplementary Material

Predicting tick presence by environmental risk mapping

Arno Swart*[1], Adolfo Ibañez-Justicia[2], Jan Buijs[3], Sip E. van Wieren[4], Tim R. Hofmeester[4], Hein Sprong[1], Katsuhisa Takumi[1]

1 Centre for Infectious Disease Control, National Institute for Public Health and the Environment, Bilthoven, The Netherlands

2 Centre for Monitoring of Vectors, Wageningen, The Netherlands

3 Public Health Service of Amsterdam, Amsterdam, The Netherlands

4 Resource Ecology Group, Wageningen UR, Wageningen, The Netherlands

*** Correspondence:** Arno Swart, Centre for Infectious Disease Control, RIVM (National Institute for Public Health and the Environment), P.O. Box 1, 3720 BA, Bilthoven, The Netherlands. arno.swart@rivm.nl

9. Supplementary Figures and Tables

The figures 1 to 6 on the following pages show additional results of scenarios that are too extensive for the main text. Each figure presents an alternative scenario, according to the following table.

Figure	Tick presence threshold	No prediction threshold	EM, p-value	Tickbite, p-value
1	0	0.95	8.9×10^{-16}	3.5×10^{-12}
2	5	0.95	7.6×10^{-11}	1.6×10^{-13}
3	0	0.80	1.1×10^{-17}	5.8×10^{-14}
4	5	0.80	1.9×10^{-14}	2.7×10^{-13}
5	0	0.00	3.0×10^{-20}	1.5×10^{-15}
6	5	0.00	1.2×10^{-17}	1.6×10^{-14}

The tick presence threshold indicates the number ticks below which we consider a point as ‘absence’ point. The no-prediction threshold is the value of the chi-square criterion (see main text) below which we consider a point as informative. Points below this value are not included in the figure, nor in the regression with the incidence data. Thus, a threshold of 0.95 is very strict, while a threshold of 0.00 is no threshold at all. The above table further gives the p-value for the intercept of the linear regression, of hazard against incidence.

For each figure we show six panels,

A. Prediction

B. Prediction averaged per municipality

C. EM incidence per municipality

D. The residual, normalized EM incidence minus Prediction averaged per municipality

E. Tick bite incidence per municipality

F. The residual, normalized tick bite incidence minus Prediction averaged per municipality

Note how panels C and E are equal for each figure. We present them anyway, for reference within one figure.

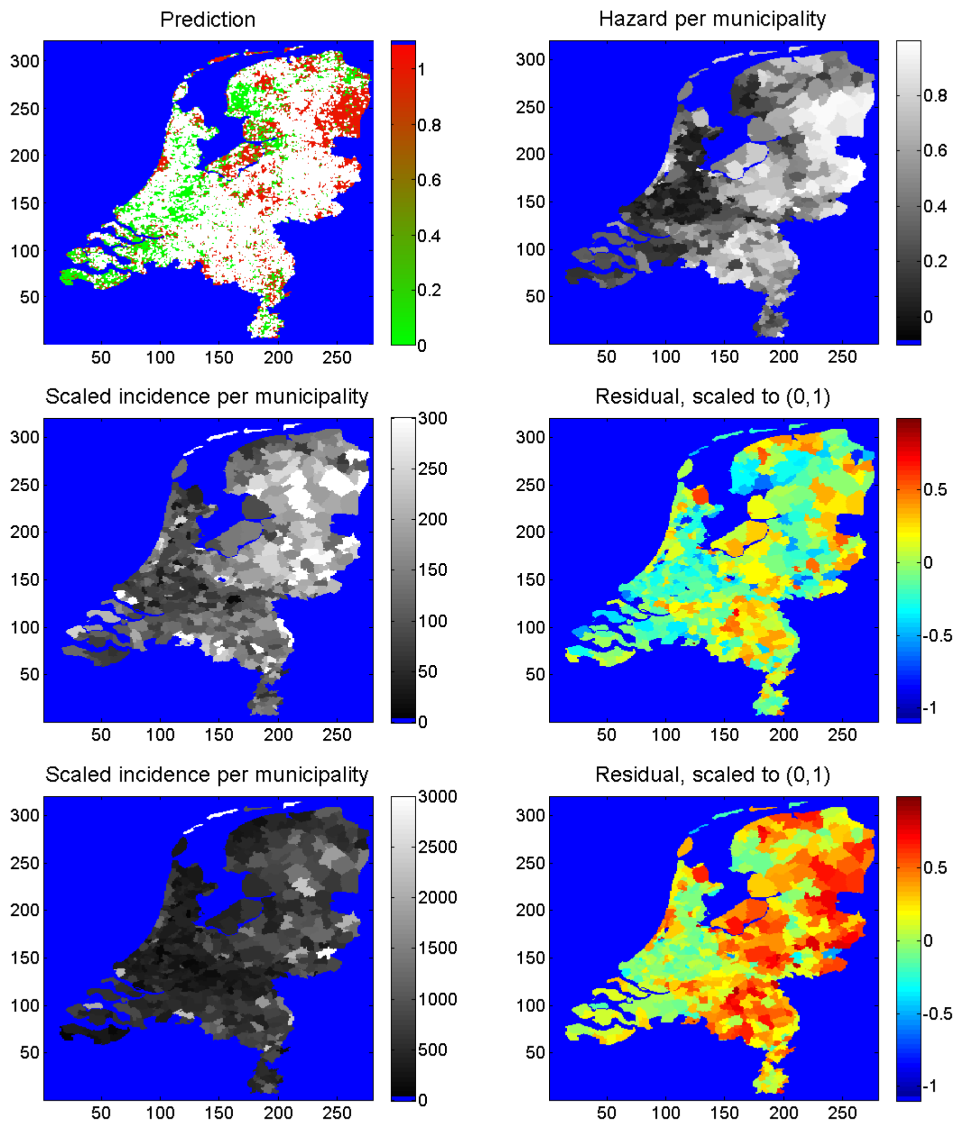

Figure S1 - Panels for a tick presence threshold of 0, a no prediction threshold of 0.95.

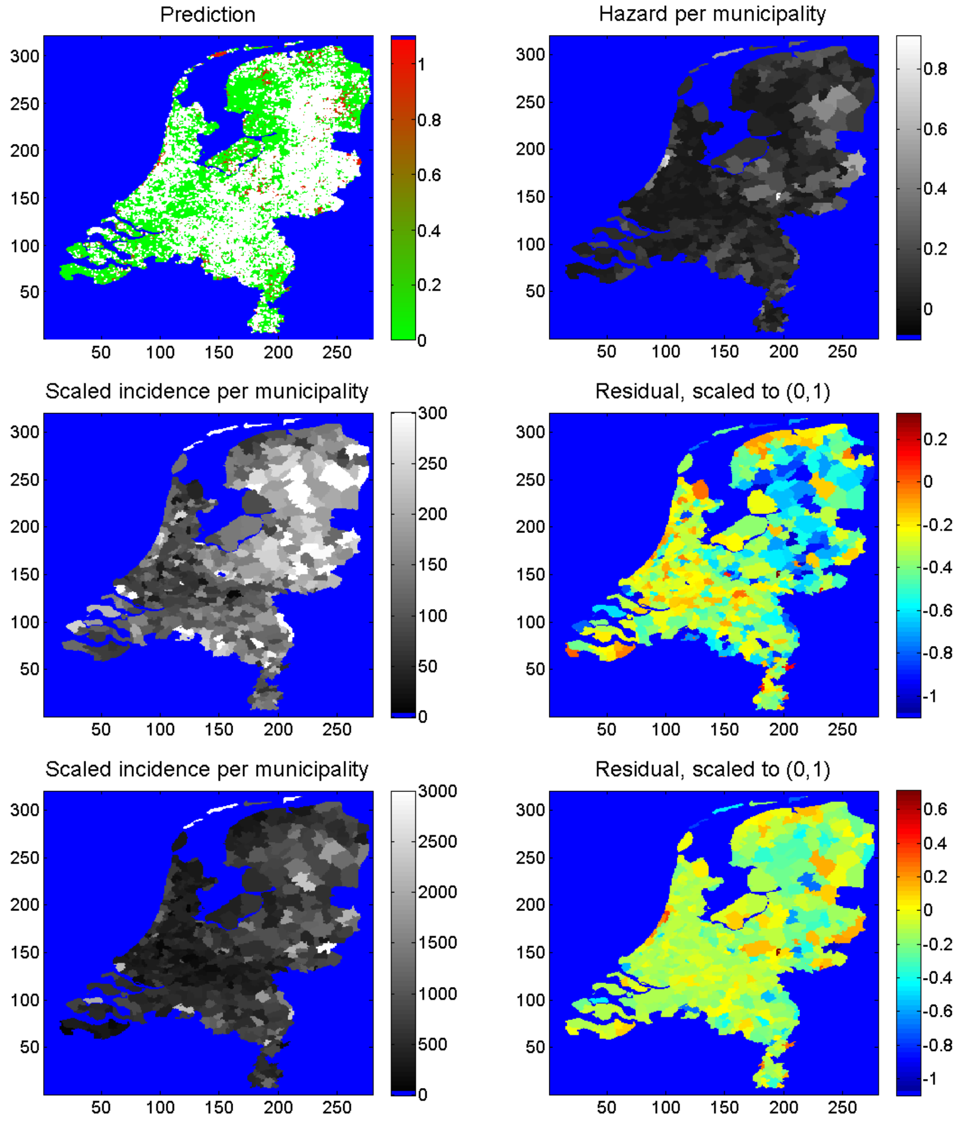

Figure S2 - Panels for a tick presence threshold of 5, a no prediction threshold of 0.95.

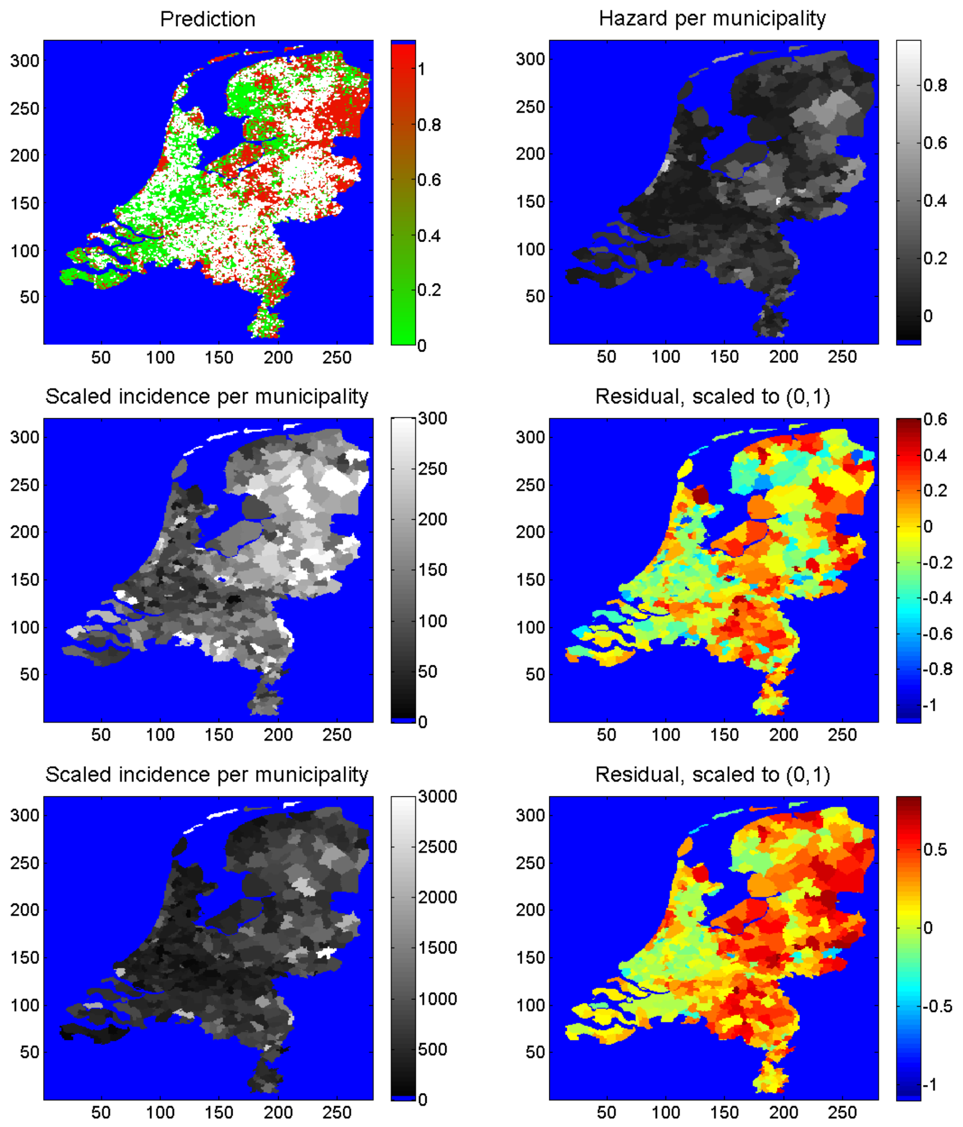

Figure S3 - Panels for a tick presence threshold of 0, a no prediction threshold of 0.80.

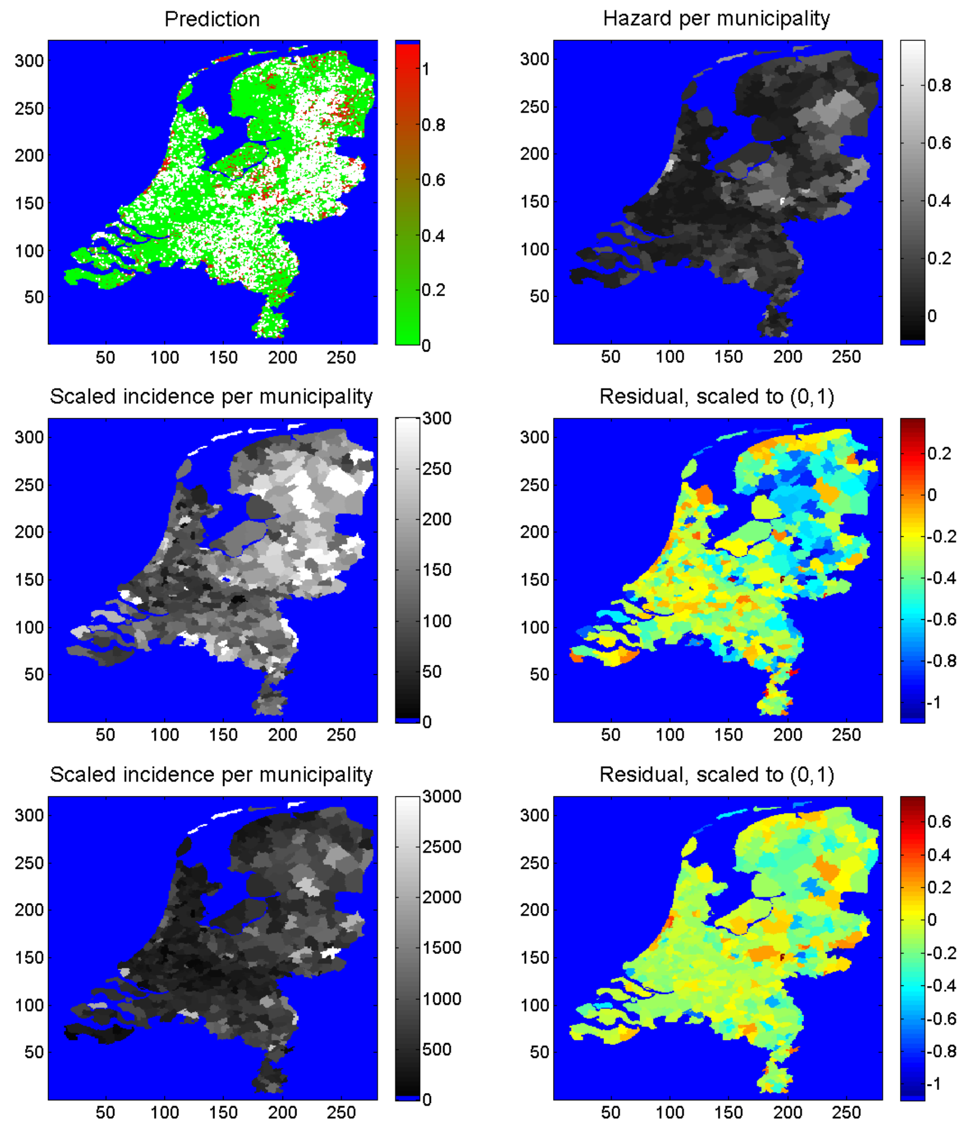

Figure S4 - Panels for a tick presence threshold of 5, a no prediction threshold of 0.80.

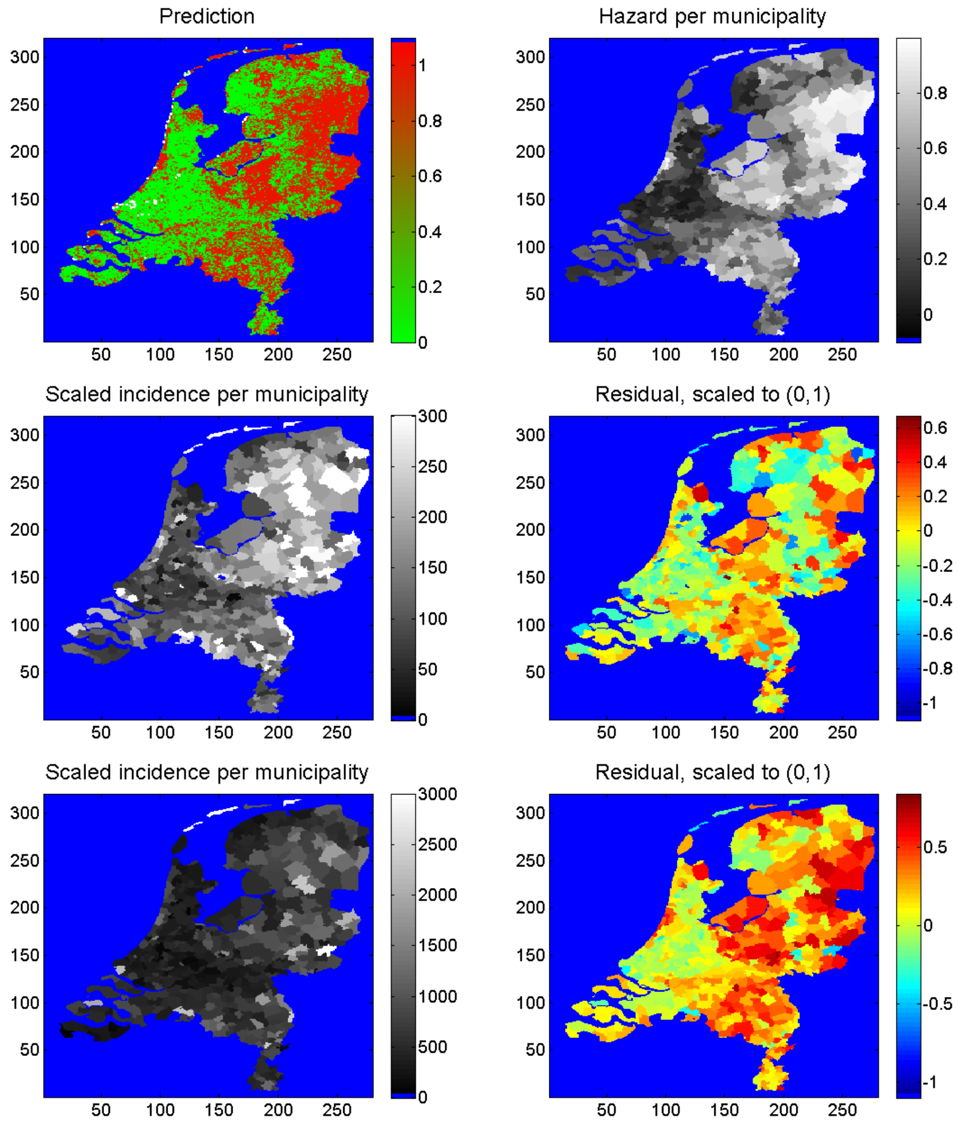

Figure S5 - Panels for a tick presence threshold of 0, a no prediction threshold of 0.0.

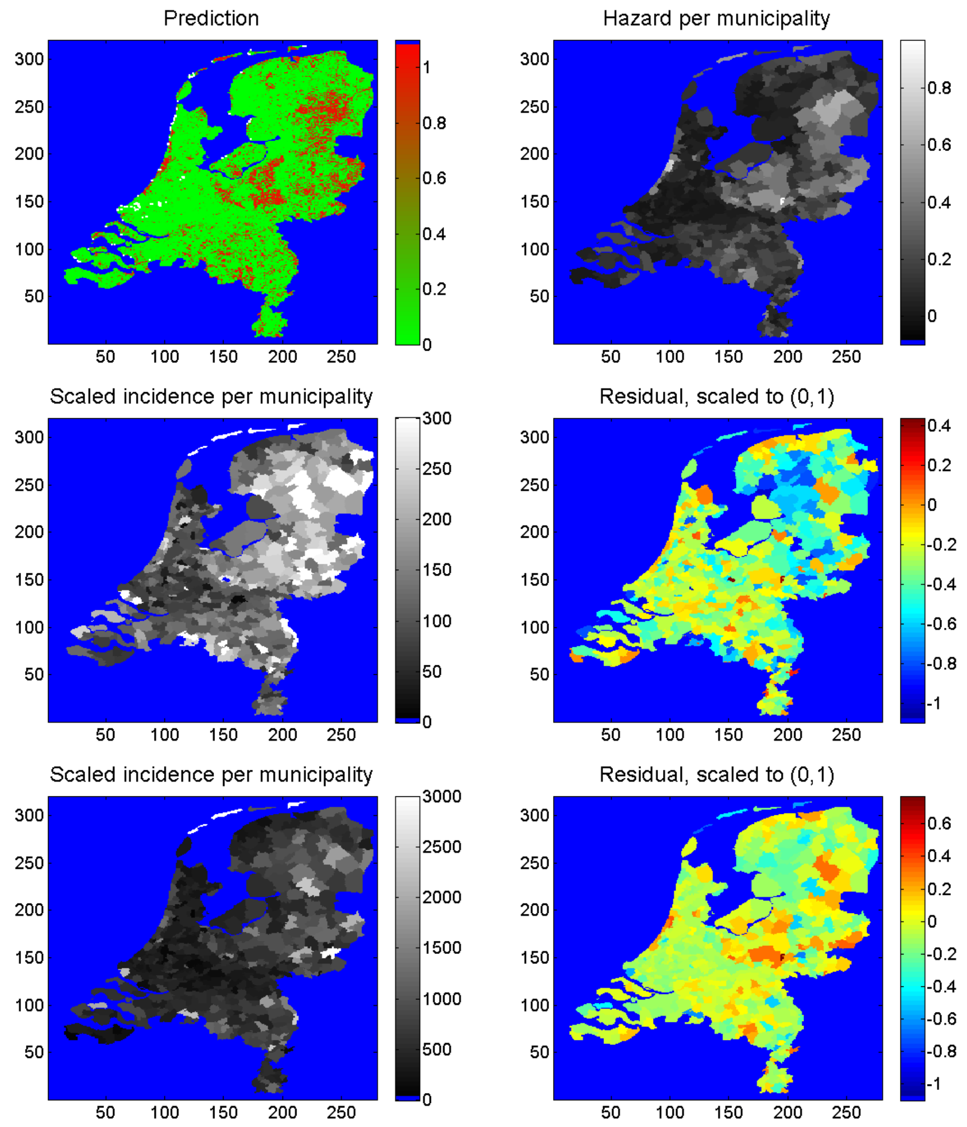

Figure S6 - Panels for a tick presence threshold of 5, a no prediction threshold of 0.0
